# Induction of miR-665-3p Impairs the Differentiation of Myogenic Progenitor Cells by Regulating the TWF1-YAP1 Axis

**DOI:** 10.3390/cells12081114

**Published:** 2023-04-08

**Authors:** Mai Thi Nguyen, Wan Lee

**Affiliations:** 1 Department of Biochemistry, Dongguk University College of Medicine, 123 Dongdae-ro, Gyeongju 38066, Republic of Korea; 2 Channelopathy Research Center, Dongguk University College of Medicine, 32 Dongguk-ro, Ilsan Dong-gu, Goyang 10326, Republic of Korea

**Keywords:** actin dynamics, twinfilin-1, miR-665-3p, obesity, myogenesis, differentiation, YAP1, F-actin, proliferation

## Abstract

Actin dynamics are known to orchestrate various myogenic processes in progenitor cells. Twinfilin-1 (TWF1) is an actin-depolymerizing factor that plays a crucial role in the differentiation of myogenic progenitor cells. However, little is known about the mechanisms underlying the epigenetic regulation of TWF1 expression and impaired myogenic differentiation in the background of muscle wasting. This study investigated how miR-665-3p affects TWF1 expression, actin filaments’ modulation, proliferation, and myogenic differentiation in progenitor cells. Palmitic acid, the most prevalent saturated fatty acid (SFA) in food, suppressed TWF1 expression and inhibited the myogenic differentiation of C2C12 cells while increasing the level of miR-665-3p expression. Interestingly, miR-665-3p inhibited TWF1 expression by targeting *TWF1* 3′UTR directly. In addition, miR-665-3p accumulated filamentous actin (F-actin) and enhanced the nuclear translocation of Yes-associated protein 1 (YAP1), consequently promoting cell cycle progression and proliferation. Furthermore, miR-665-3p suppressed the expressions of myogenic factors, i.e., MyoD, MyoG, and MyHC, and consequently impaired myoblast differentiation. In conclusion, this study suggests that SFA-inducible miR-665-3p suppresses TWF1 expression epigenetically and inhibits myogenic differentiation by facilitating myoblast proliferation via the F-actin/YAP1 axis.

## 1. Introduction

Skeletal muscle is the most abundant body component and serves diverse functions, including force generation, locomotion, energy storage, and metabolism [1]. Homeostasis in skeletal muscle requires the capability of myogenesis, which is essential for the development, growth, and regeneration of multinucleated myofibers [2]. Skeletal myogenesis is a finely coordinated multistage process that comprises progenitor cell proliferation, myogenic factor activation, differentiation, and fusion to form myotubes [2]. Hence, impaired skeletal myogenesis results in muscle wasting, characterized by an overall decline in muscle mass, strength, quality, and regeneration ability [3]. In recent years, several studies have demonstrated that the excessive consumption of saturated fatty acids (SFA) leads to ectopic fat accumulation in muscle tissues and provokes lipotoxicity, such as mitochondrial dysfunction, oxidative stress, apoptosis, and eventually results in muscle wasting [4,5]. However, it still remains unclear which mechanisms underlie the impairment of the differentiation of myogenic progenitor cells and the loss of muscle tissue caused by SFA.

Cytoskeleton remodeling, such as assembling, disassembling, and rearranging cytoskeletal proteins, has been shown to orchestrate various myogenic transcriptional programs in progenitor cells [6,7]. Actin is a critical regulator in cytoskeleton remodeling because it has the ability to polymerize and depolymerize dynamically [6,8]. Recently, actin-binding proteins have been known to play a significant role in mechanotransduction, in which mechanical and physical changes are converted into biochemical signals, thus triggering cellular responses related to the cell cycle and proliferation [8,9,10]. Due to the advances in mechanotransduction research, a growing body of evidence suggests that actin-binding proteins regulate Yes-associated protein 1 (YAP1) by modulating the conformational dynamics between globular actin (G-actin) and filamentous actin (F-actin) [11,12,13]. YAP1 is a transcriptional coactivator in the Hippo signaling pathway that regulates a variety of cellular functions, including the cell cycle, proliferation, differentiation, and survival [14]. Therefore, F-actin accumulation in the cells has been shown to facilitate cell proliferation via YAP1 activation as a mechanotransduction mechanism [15]. From this point of view, it is interesting that Twinfilin-1 (TWF1), an actin filament depolymerizing factor, was recently demonstrated to play an essential role in myogenic differentiation [16]. The knockdown of TWF1 accumulated F-actin and enhanced cell proliferation via YAP1 activation, thereby impairing myogenic differentiation in myoblasts [16]. Despite this, the epigenetic regulation and role of TWF1 in myogenesis remain unknown under conditions related to muscle wasting, such as obesity, oxidative stress, and inflammation.

MicroRNAs are a family of non-coding RNAs that suppress gene expression by binding to the 3′UTR of target mRNAs [17] and are known to play a significant role in most biological processes, including cell proliferation, cell cycle coordination, and differentiation [18,19,20,21,22]. Over the last decade, it has been demonstrated that certain miRNAs affected by SFA or obesity are involved in the etiology of muscle wasting [23,24,25]. Furthermore, several studies have linked the dysregulation of specific miRNAs with various myopathies [26,27]. However, the mechanisms by which SFA-induced miRNAs impair skeletal myogenesis remain largely unknown. Previous studies have revealed that miR-665-3p, a member of the DLK1-DIO3 miRNA cluster, is upregulated in obesity [28,29]. Based on miRNA profiling of the percutaneous muscle from men and women, miR-665-3p is increased significantly in obese subjects compared to lean subjects (GSE99891 from the public Gene Expression Omnibus). Similarly to previous studies, we also found an upregulation of miR-665-3p in the liver of diet-induced obese mice [30] and HepG2 cells treated with palmitic acid (PA) [31]. These results suggest that miR-665-3p is SFA-inducible and obesity-associated. In a recent study, miR-665-3p levels in differentiating C2C12 myocytes were significantly lower than in undifferentiated proliferating myoblasts (GSE29286) [32]. Moreover, miR-665-3p has been reported to be significantly elevated by senescence, mitochondrial dysfunction, and oxidative stress, which are all positively associated with muscle wasting [33,34,35,36]. Interestingly, a bioinformatic analysis using a TargetScan analysis suggested that TWF1 is a potential target of miR-665-3p. However, the significance and mechanism of miR-665-3p in the regulation of TWF1 expression and myogenic differentiation have not been investigated.

The present study revealed that SFA regulates the expression of TWF1 and miR-665-3p inversely in myoblasts and confirms TWF1 as an authentic target of miR-665-3p. As TWF1 is an essential factor for myoblast differentiation, we demonstrated the role of miR-665-3p in myogenic factors’ expression and myogenic differentiation in progenitor cells. Mechanistically, we showed how miR-665-3p enhanced myoblast proliferation through F-actin formation and YAP1 activation. Therefore, this study underscores the role played by miR-665-3p in myogenesis and suggests a possible mechanism for miRNA-mediated muscle wasting in the background of obesity.

## 2. Materials and Methods

### 2.1. Cell Culture, Differentiation, and Palmitic Acid (PA) Treatment

C2C12 cells (a murine myogenic progenitor cell line) were purchased from ATCC (Manassas, VA, USA) and incubated in a growth medium (GM) consisting of DMEM supplemented with FBS (10%) and penicillin-streptomycin (1%) (Gibco, Carlsbad, CA, USA) at 37 °C in a humidified 5% CO_2_ atmosphere. For transfection, cells were seeded in 35 mm plates at 1.3 × 10^5^ cells/dish for 24 h and then transfected with Lipofectamine 2000 (Invitrogen, Carlsbad, CA, USA), according to the manufacturer’s instructions. For myogenic differentiation, the GM was replaced with a differentiation medium (DM; 2% horse serum in DMEM with 1% penicillin-streptomycin) (Gibco) when the cell population reached 80–90% confluence. PA conjugated to bovine serum albumin (BSA) was prepared as previously described [37], and cells were treated with 100 μM PA or vehicle in GM for 24 h before analysis or inducing differentiation.

### 2.2. Transfection of Oligonucleotides

C2C12 cells were transfected with scrambled control RNA (scRNA), small interfering RNA molecules against TWF1 (siTWF1), miR-665-3p mimic, or miR-665 inhibitor (antimiR-665) at 200 nM (Genolution, Seoul, Republic of Korea) using Lipofectamine 2000 (Invitrogen) in FBS-free DMEM for 4 h, according to the manufacturer’s instructions. Afterward, the media was replaced with fresh GM. Oligonucleotide sequences used in this study are provided in Table S1.

### 2.3. qRT-PCR

Qiazol reagent (Qiagen, Hilden, Germany) was used to isolate total RNAs from C2C12 cells. RNA quality and purity were confirmed using a miRNeasy Mini Kit (Qiagen), and RNA concentrations were determined using a UV-1700 PharmaSpec spectrophotometer (Shimadzu, Kyoto, Japan). cDNA was prepared with reverse transcription using a miScript II RT Kit (Qiagen) and subjected to *q*RT-PCR in a Light Cycler 480 (Roche Applied Science, Penzberg, Germany) using SYBR Green I (Promega, Madison, WI, USA). Reaction conditions and the sequences of primers used for *q*RT-PCR are listed in Table S2. Relative gene expressions were calculated using the 2^−ΔΔCt^ method, and U6 was used as the internal control.

### 2.4. Dual-Luciferase Assay

The mouse *TWF1* 3′UTR containing a potential binding site of miR-665-3p was synthesized using Pfu DNA polymerase (Invitrogen) and inserted into the pmirGLO vector (Promega) to generate a wild-type plasmid (TWF1*wt*). The mutagenesis was carried out using overlapping PCR containing the mutated target sequence of miR-665-3p and constructed into the pmirGLO vector to generate a mutated plasmid (TWF1*mut*) following the manufacturer’s instructions. The primer sequences used for cloning and site-directed mutagenesis are provided in Table S2. For luciferase analysis, C2C12 cells (1 × 10^4^ per well) were grown in a 12-well plate for 24 h before co-transfection with 0.5 μg pmirGLO luciferase vector containing either TWF1*wt* or TWF1*mut* and miR-665 mimic or scRNA control. The Dual-Luciferase Reporter Assay System 100 kit (Promega) and the Sirius L Single Tube Luminometer system (Berthold Technology, Bad Wildbad, Germany) were used to determine Firefly and Renilla luciferase activities after 24 h of transfection. The relative Renilla/Firefly ratios determined the luciferase activities.

### 2.5. Preparation of Cytoplasmic and Nuclear Fractions

Cell fractions were extracted using NE-PER^TM^ Nuclear and Cytoplasmic Extraction Reagents (Thermo Fisher Scientific, Waltham, MA, USA) to obtain separate cytoplasmic and nuclear fractions. Briefly, C2C12 cells were collected using trypsin EDTA (Gibco), washed three times with pre-cold PBS, and centrifuged at 3000 rpm for 5 min at 4 °C. Pellets were suspended in 100 µL CER I and incubated on ice for 30 min, and 5.5 µL CER II was added, incubated for 1 min, and centrifuged. Supernatants were retained as cytoplasmic fractions, and pellets were resuspended in 50 µL of ice-cold NER solution, vortexed 3 times every 10 min, and centrifuged. Supernatants were collected as nuclear fractions. The protein of cytoplasmic and nuclear fractions was subjected to immunoblots.

### 2.6. Immunoblot Analysis

Total protein was extracted from C2C12 cells using a lysis buffer containing 0.2 mM PMSF, 2% Triton-X, and 1% phosphatase inhibitor cocktail II (Sigma-Aldrich, St. Louis, MO, USA). Protein concentrations were determined using the Bradford assay with a UV-1700 PharmaSpec spectrophotometer (Shimadzu). The protein samples (20 µg/well) were separated by electrophoresis and transferred onto nitrocellulose membranes (Amersham, Braunschweig, Germany). After blocking with 5% skim milk in TTBS solution (1% Tween 20 in TBS) for 1 h, the membranes were incubated with primary antibodies (Table S3) overnight at 4 °C, washed with TTBS (6 × 5 min), and developed with a secondary antibody (1:10,000 dilution). The blots were then visualized using Femto reagent (Thermo Fisher Scientific, Waltham, MA, USA) and the analytical scanning system Fusion Solo (Vilber, Marne-la-Vallée, France), and the densities of blots were analyzed with Evolution Capt software (Vilber).

### 2.7. Immunofluorescence Analysis

C2C12 cells were transfected with scRNA, siTWF1, miR-665-3p mimic, or antimiR-665 and differentiated for five days. For immunofluorescence analysis, cells were washed with PBS, fixed with 4% paraformaldehyde, permeabilized with 0.3% Triton X-100, and blocked in PBS containing 3% BSA for 2 h. The cells were incubated overnight with a myosin heavy chain (MyHC) antibody (1:100 dilution) and then with a secondary antibody (Alexa 488, Invitrogen) for 1 h. For F-actin staining, cells were transfected for 24 h, fixed, permeabilized, and incubated with FITC-coupled phalloidin (P5282, Sigma-Aldrich, St. Louis, MO, USA) at room temperature, as previously described [13]. Nuclei were visualized by counterstaining with Hoechst 33,342 (Invitrogen), and fluorescent images were captured randomly using a Leica fluorescence microscope (Microsystems, Mannheim, Germany). All measurements were obtained from at least five separated areas per experiment for at least three independent experiments. Images were analyzed using ImageJ software for myotube widths, MyHC-positive areas, and myotube numbers. Differentiation and fusion indices were calculated as previously described [16].

### 2.8. Cell Proliferation Assay

The Click-iT™ EdU (5-ethynyl-2′-deoxyuridine) Cell Proliferation Kit (Invitrogen) was used to quantify cell proliferation ability. In brief, cells were incubated with EdU (10 µM) in GM for 4 h and then fixed with 4% formaldehyde, as described above. Further, 300 µL of Click-iT^®^ reaction cocktail was added, and nuclei were counterstained for 15 min with Hoechst 33,342 (Invitrogen). Images were obtained using a Leica fluorescent microscope. To calculate EdU-positive cell proportions and total cell numbers, cells were counted in randomly selected images from three independent experiments.

### 2.9. Flow Cytometry

Transfected cells were collected into a 2 mL tube using trypsin EDTA (Gibco) and centrifuged at 3000 rpm for 5 min at 4 °C. Cell pellets were resuspended in 70% ethanol (1 mL), incubated at 4 °C, fixed overnight, washed three times, and stained with a Cell Cycle kit (0.5 mL, C03551; Beckman Coulter, Brea, CA, USA) for 20 min in the dark. Cell cycles were analyzed using a CytoFLEX unit (Beckman Coulter).

### 2.10. Cell Viability

C2C12 cells were grown in 96-well plates at 10^3^ cells/well and treated with PA or vehicle for 24 h. Then, 10 μL Quanti-maxTM WST-8 Cell viability Assay Kit solution (BioMax, Seoul, Republic of Korea) in 100 μL of GM was added to each well and continuously incubated for 4 h at under 37 °C. Finally, a microplate reader (Model 680, Bio-rad) was used to determine the absorbance at a wavelength of 450 nm.

### 2.11. Animal Experiment

The C57BL/6N strain from male mice aged six wks (OrientBio, Seongnam, Republic of Korea) were divided randomly into two groups. One group was fed a high-fat diet (HFD) containing 60% fat from Dyets Inc (Bethlehem, PA, USA), while the other group received a normal-fat diet (NFD) with 11% fat from Purina (Wilkes-Barre, PA, USA). The composition of each diet is described in Table S4. The mice were provided with food and water ad libitum, and after 14 wks on their respective diets, they were euthanized, and skeletal muscle tissues (gastrocnemius muscle) were collected to analyze the expressions of TWF1 and miR-665-3p. The Animal Use and Care Committee of Dongguk University approved the in vivo experimental protocol with the approval number IACUC-2020-007.

### 2.12. Database and Statistical Analysis

MiRNA microarray datasets were obtained from the public Gene Expression Omnibus (GEO) database (www.ncbi.nlm.nih.gov/geo). The binding sites of miRNAs on *TWF*1 3′UTR were predicted using publicly available bioinformatics databases (Pictar: pictar.mdc-berlin.de and TargetScan: www.targetscan.org). Results are presented as the means ± SEM of at least three independent experiments. The analysis was conducted using the one-way independent Student’s *t*-test for unpaired data.

## 3. Results

### 3.1. PA Inhibited TWF1 Expression but Upregulated miR-665-3p Expression

**Figure 1:**
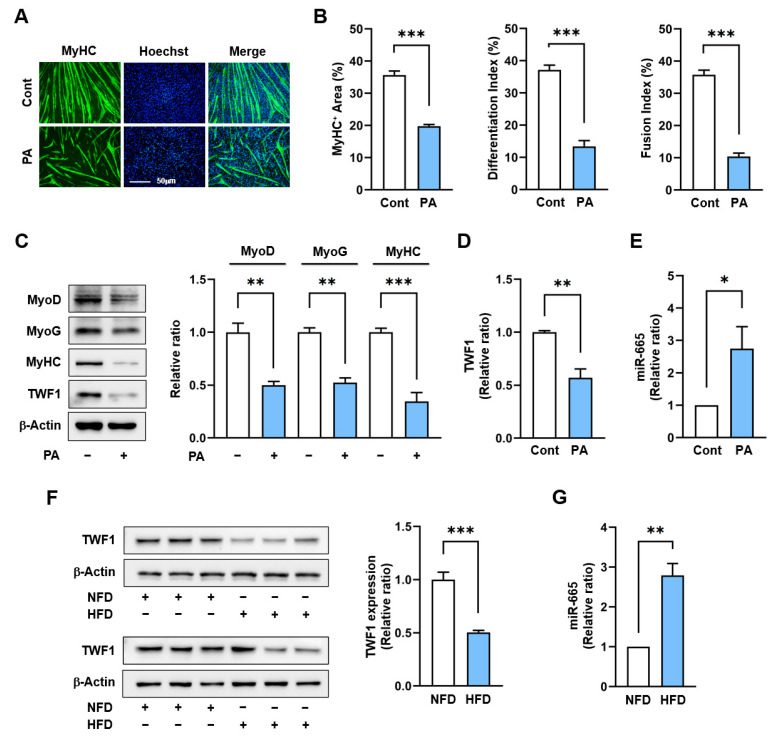
PA inhibited C2C12 differentiation and upregulated miR-665-3p expression. C2C12 cells were treated with PA (100 μM) or vehicle control for 24 h before inducing differentiation. (**A**) After differentiation for five days, immunofluorescence staining with MyHC (green) was used to analyze myogenic differentiation. Nuclei were counterstained with Hoechst 33,342 (blue). Scale bar: 50 μm. (**B**) Myotube formation was quantified based on the results of (**A**) using MyHC-positive area, differentiation indices, and fusion indices. (**C**,**D**) The expressions of myogenic factors and TWF1 were analyzed on day three of differentiation. Expression levels were normalized versus β-actin. (**E**) The expression of miR-665-3p was analyzed 24 h after PA treatment by *q*RT-PCR and normalized to U6. (**F**) The representative immunoblots (6 mice/group) of TWF1 in the gastrocnemius muscle of NFD-fed mice and HFD-fed mice are shown. (**G**) The expression of miR-665-3p in the gastrocnemius muscle of NFD-fed mice (open column) and HFD-fed mice (closed column) was analyzed by *q*RT-PCR and normalized to U6. The values are expressed as the relative ratio, where the intensity of the vehicle control was set to one. Data are presented as means ± SEMs (*n* = 3 except mice experiment (*n* = 6); * *p* < 0.05; ** *p* < 0.01; *** *p* < 0.001 vs. control or NFD).

To investigate the role of PA on myoblast differentiation, we treated C2C12 cells with PA (100 μM) for 24 h and then allowed them to differentiate for five days. As reported previously, a concentration of 100 μM PA inhibited myoblast differentiation without affecting cell viability [19]. Based on the results of the cell proliferation analysis (Figure S1) and the cell nucleus count evaluation (Figure S2) in this study, it was determined that this concentration did not affect cell proliferation and, thus, was applied in the experiment. Immunostaining for MyHC on differentiation day five showed that PA treatment resulted in a decrease in MyHC-positive areas and exhibited shorter myotubes and fewer nuclei than vehicle-treated controls (Figure 1A,B). In addition, PA dramatically reduced levels of myogenic regulatory proteins, such as MyoD and MyoG (Figure 1C). These results suggest that PA suppresses the expressions of myogenic factors and inhibits myotube formation in myoblasts. Since TWF1 is required for myogenic differentiation [16] and miR-665-3p levels are increased in obesity [28,29,31], we next investigated whether TWF1 and miR-665-3p expressions are affected by PA treatment in C2C12 cells. Notably, PA reduced TWF1 protein expression by approximately 60% (Figure 1C,D), whereas it increased miR-665-3p levels more than 2.5-fold compared to the vehicle controls (Figure 1E). These observations suggested that PA inhibits myogenic differentiation and negatively regulates the expression of TWF1 while inducing miR-665-3p expression in C2C12 myoblasts. Furthermore, to examine the effect of obesity on TWF1 and miR-665-3p expression in the skeletal muscle, we utilized HFD-induced obese mice as described in the Section 2. The HFD-fed mice showed a significant increase in body weight compared to the NFD-fed control group, as shown in Figure S3. The results revealed that the HFD-induced obese mice had decreased TWF1 expression and increased miR-665-3p expression in the gastrocnemius muscle (Figure 1F,G), indicating that obesity may negatively affect the expression of TWF1 in skeletal muscle while increasing miR-665-3p levels.

### 3.2. MiR-665-3p Directly Targeted The 3′UTR of TWF1

**Figure 2:**
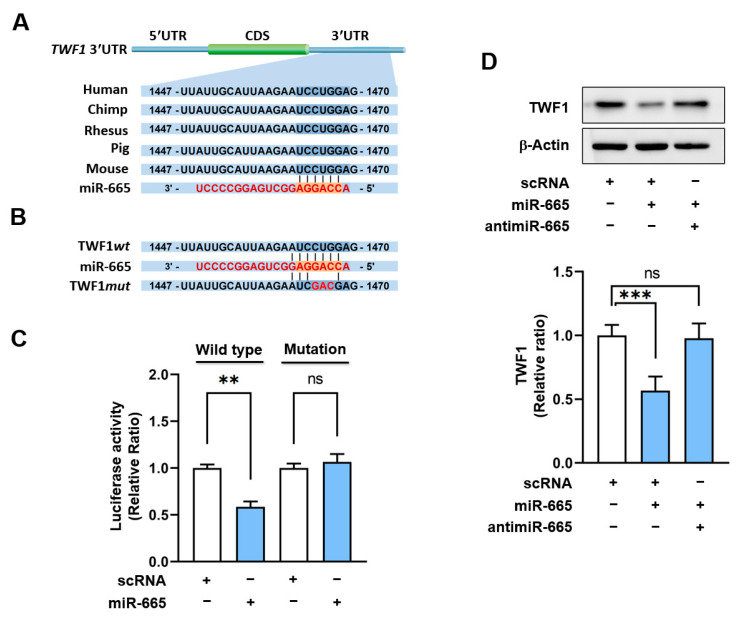
MiR-665-3p suppressed TWF1 expression. (**A**) Sequence conservation analysis of miR-665-3p among different species was conducted using TargetScan and Pictar. (**B**) Schematic diagram of dual-luciferase reporter plasmids pmirGLO-TWF1 3′UTR-wild type (TWF1*wt*) with binding sites for the seed sequence of miR-665-3p and its mutant (TWF1*mut*). (**C**) Dual luciferase activity was determined 24 h after transfection. (**D**) Representative immunoblots of TWF1 expression in 200 nM of miR-665-3p, antimir-665, or scRNA-transfected cells after transfection for 24 h. Results are expressed as the means ± SEMs of intensity ratios versus scRNA controls (*n* = 3; ** *p* < 0.01; *** *p* < 0.001 vs. scRNA). ns: no significance.

To determine whether miR-665-3p suppresses TWF1 expression, we explored its potential target genes using the miRNA target prediction algorithms, such as PicTar and TargetScan. Comparative sequencing analysis showed that the nucleotide sequence (TCCTGGA) of the 3′UTR of *TWF1* is complementary to the seed sequence of miR-665-3p, which is evolutionarily conserved in many species, including human, mouse, and rabbit (Figure 2A). Therefore, to determine whether the 3′UTR of *TWF1* includes an authentic binding site for miR-665-3p, we cloned *TWF1* 3′UTR containing the miR-665-3p seed binding site (wild-type; TWF1*wt*) or a three-base mutation (TWF1*mut*) at the binding site into a pmirGLO dual-luciferase reporter plasmid (Figure 2B). These constructs were then cotransfected with miR-665-3p mimic or scRNA into C2C12 cells and luciferase activity was determined after 24 h of transfection. Cotransfection of the miR-665-3p mimic with the TWF1*wt* construct decreased luciferase activity by 45% vs. scRNA controls (Figure 2C). However, cotransfection of the miR-665-3p mimic with the TWF1*mut* construct did not decrease luciferase activity (Figure 2C). Next, to confirm that TWF1 is a valid target of miR-665-3p, we assessed TWF1 protein levels after transfecting C2C12 cells with the miR-665-3p mimic, antimiR-665, or scRNA. As was expected, the miR-665-3p mimic significantly reduced TWF1 protein levels vs. scRNA controls (Figure 2D), but inhibition of miR-665-3p using 2’-O-methyl antisense inhibitors of miR-665-3p (antimiR-665) completely prevented this reduction in endogenous TWF1 protein levels (Figure 2D). Therefore, these results indicate that miR-665-3p negatively regulated TWF1 expression by binding directly to its 3′UTR.

### 3.3. MiR-665-3p Augmented F-Actin and Nuclear YAP1 Levels

**Figure 3:**
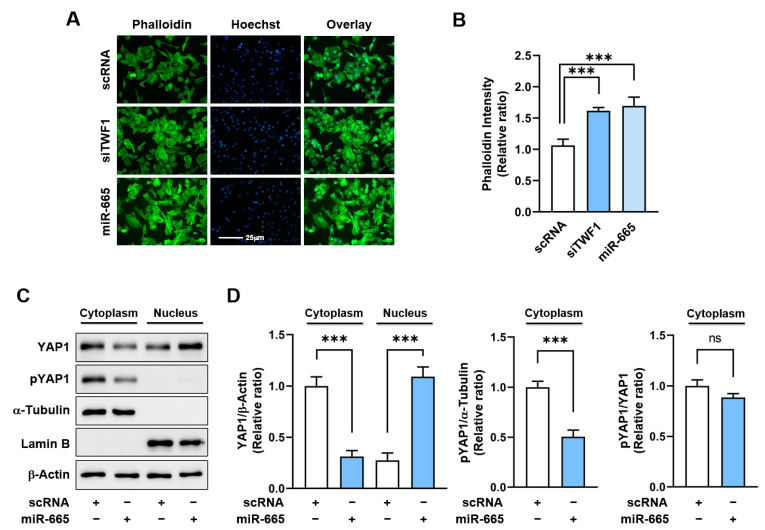
MiR-665-3p promoted F-actin accumulation and the nuclear localization of YAP1. C2C12 cells were transfected with 200 nM scRNA, siTWF1, or miR-665-3p mimic for 24 h. (**A**,**B**) FITC-Phalloidin staining was used to analyze F-actin formation (green). Nuclei were counterstained with Hoechst 33,342 (blue). Scale bar: 25 μm. (**C**) Representative immunoblots of YAP1 and phosphor-YAP1 (pYAP1) in the nuclear and cytoplasmic fractions. The quality of subcellular fractionation was confirmed by Lamin B and α-Tubulin, which were used as nuclear and cytoplasmic markers, respectively. (**D**) Densitometry results of YAP1 and pYAP1 expression were normalized versus designated molecules, such as β-Actin, α-Tubulin and YAP1, respectively. Results are expressed as the means ± SEMs of intensity ratios versus scRNA controls (*n* = 3; ***, *p* < 0.001 vs. scRNA). ns: no significance.

Because the TWF1 knockdown in myoblasts was previously shown to facilitate cell proliferation by augmenting F-actin and nuclear YAP1 levels [16], we investigated whether miR-665-3p overexpression leads to F-actin accumulation and YAP1 activation. Figure 3A,B show that F-actin levels were significantly elevated by approximately 50% in the C2C12 cells transfected with the miR-665-3p mimic or TWF1 siRNA (siTWF1) vs. scRNA controls. Since immunoblot analysis exhibited no difference between these treatments in terms of total actin levels, increases in F-actin levels appeared to be due to impaired actin depolymerization resulting from TWF1 inhibition by miR-665-3p. This result supports the hypothesis that miR-665-3p inhibits actin depolymerization by inhibiting TWF1 and thereby promoting F-actin accumulation. Actin stress fibers have been shown to prevent the phosphorylation of the transcriptional coactivator YAP1, which enables the nuclear translocation of YAP1, and, thus, YAP1 binding to the TEA domain family and activation of the transcriptional factors involved in cell proliferation [14,38]. Hence, we next assessed whether miR-665-3p might stimulate the nuclear translocation of YAP1 in myoblasts by quantifying its phosphorylation (Y357) and nuclear levels (Figure 3C,D). As was anticipated, myoblasts overexpressing the miR-665-3p mimic exhibited a significant decrease in cytoplasmic phosphor-YAP1 and an increase in nuclear YAP1, indicating that miR-665-3p can facilitate the nuclear translocation of YAP1.

### 3.4. MiR-665-3p Enhanced Cell Proliferation

**Figure 4:**
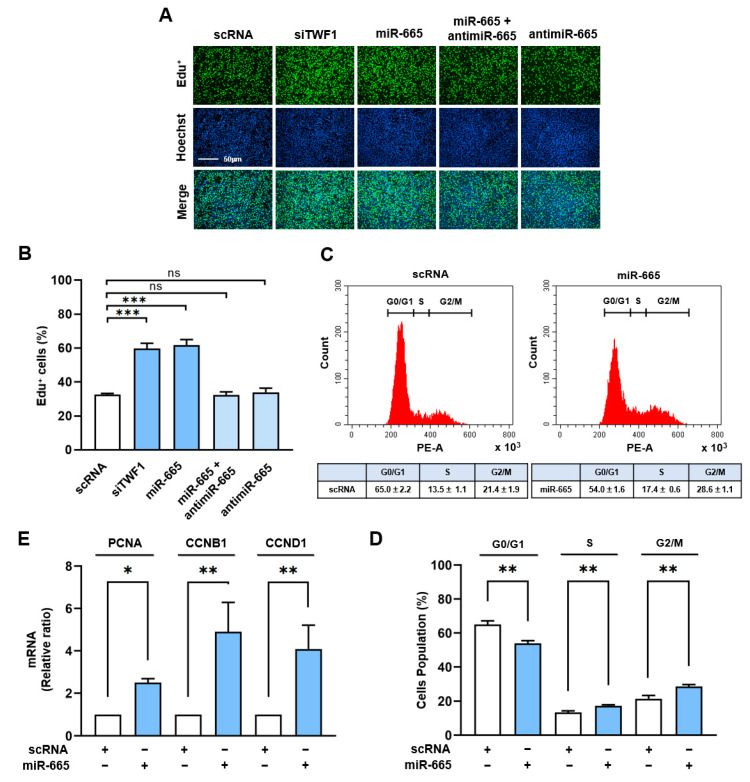
MiR-665-3p facilitated myoblast proliferation. C2C12 cells were transfected with miR-665 mimic, antimiR-665, siTWF1, or scRNA (200 nM) for 24 h. (**A**) Cell proliferation was analyzed using an EdU assay. Cells exhibiting DNA replication were labeled with EdU (green) and nuclei with Hoechst 33,342 (blue). Scale bar: 50 μm. (**B**) Proportions of EdU-positive cells were determined using ImageJ software. (**C**,**D**) Cell cycle analysis and percentages of myoblast cells in each cell cycle phase were determined 24 after transfection. (**E**) mRNA levels of CCNB1, PCNA, and CCND1 were determined using RT-*q*PCR after normalization versus U6 levels. Results are expressed as the means ± SEMs of intensity ratios versus scRNA controls (*n* = 3; * *p* < 0.05; ** *p* < 0.01; *** *p* < 0.001 vs. scRNA). ns: no significance.

Because YAP1 plays crucial roles in cell cycle progression and proliferation, we investigated whether miR-665-3p might promote these processes. A cell proliferation assay based on the nuclear incorporation of EdU (Figure 4A,B) revealed that siTWF1 significantly enhanced EdU incorporation into myoblasts by approximately 2-fold compared to scRNA controls. As was expected, the miR-665-3p mimic also drastically increased EdU incorporation by about 1.6-fold. However, cotransfection with the antimiR-665 and miR-665-3p mimic almost completely abrogated the effect of miR-665-3p on EdU incorporation, indicating that miR-665-3p stimulates myoblast proliferation. Next, we evaluated whether miR-665-3p affects the cell cycle. Flow cytometry showed that the miR-665-3p mimic significantly increased the percentage of cells in the S and G2/M phases and decreased the percentage in the G0/G1 phase (Figure 4C,D), thus demonstrating that miR-665-3p facilitates cell cycle progression. To further confirm this role of miR-665-3p, we examined the effects of miR-665-3p on the expressions of CCNB1, CCND1, and PCNA, which are known to promote cell proliferation and cell cycle progression [39]. As shown in Figure 4E, the expressions of these genes were significantly higher in the miR-665-3p mimic-transfected cells than in the scRNA-transfected controls. Collectively, these findings suggest that miR-665-3p promotes cell cycle progression and myoblast proliferation.

### 3.5. MiR-665-3p Inhibited The Expressions of Myogenic Transcription Factors

**Figure 5:**
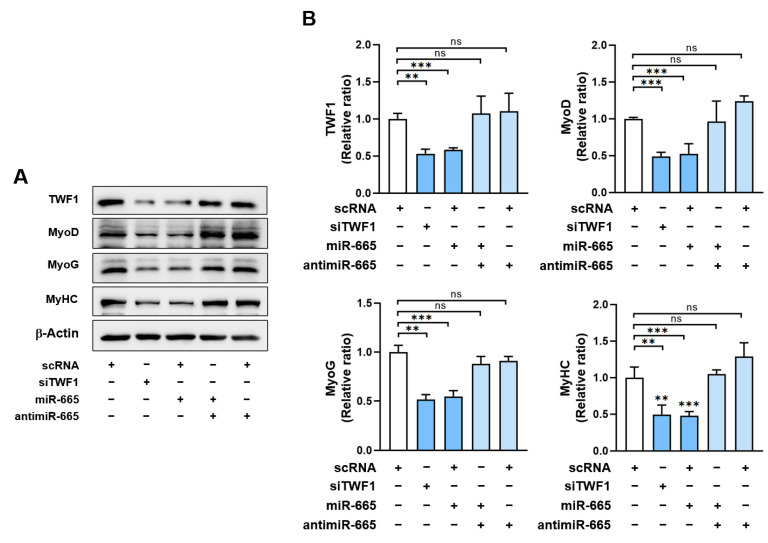
MiR-665-3p suppressed the expressions of myogenic regulator factors and TWF1. C2C12 cells were transfected with miR-665 mimic, antimiR-665, siTWF1, or scRNA (200 nM) and then differentiated for three days. (**A**) The expressions of TWF1 and myogenic factors were detected by immunoblotting. (**B**) Representative immunoblots of TWF1 and myogenic factors’ (MyHC, MyoD, and MyoG) expression as determined by densitometry and normalized versus β-actin. Results are expressed as the means ± SEMs of intensity ratios versus scRNA controls (*n* = 3; ** *p* < 0.01; *** *p* < 0.001 vs. scRNA). ns: no significance.

As the proliferation and differentiation of myoblasts are inversely related during myogenesis [2], we explored the effect of miR-665-3p on the expressions of myogenic transcription factors in myoblasts. C2C12 cells were transfected with scRNA, siTWF1, the miR-665-3p mimic, or antimiR-665, and the expressions of myogenic factors were determined on day three of differentiation. Depletion of TWF1 by siTWF1 reduced TWF1 protein levels by approximately 50% versus scRNA and significantly reduced the expressions of myogenic factors, i.e., MyoD and MyoG (Figure 5A,B). In addition, the miR-665-3p mimic transfection significantly suppressed TWF1, MyoD, and MyoG expressions versus scRNA controls, whereas co-transfection with antimiR-665 almost entirely prevented the suppression of TWF1 and myogenic factor expressions by the miR-665-3p mimic (Figure 5A,B). Since bioinformatic analysis showed the 3′UTRs of myogenic factors, i.e., MyoD, MyoG, and MyHC, lack a tentative binding site for miR-665-3p or antimiR-665, the inhibitory role of the miR-665-3p mimic on the myogenic factors’ expression was attributed to reduced TWF1 expression.

### 3.6. MiR-665-3p Impaired Myotube Formation in Myoblasts

**Figure 6:**
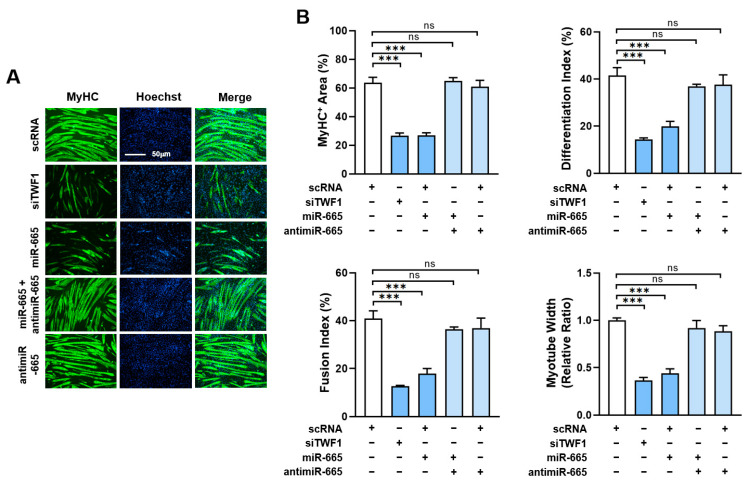
MiR-665-3p reduced myotube formation. C2C12 myoblasts were transfected with miR-665 mimic, antimiR-665, siTWF1, or scRNA control (200 μM) and then differentiated for five days. (**A**) Immunofluorescence staining (MyHC, green) was used to assess levels of differentiation of myoblasts. Nuclei were counterstained with Hoechst 33,342 (blue). Scale bar: 50 μm. (**B**) Myotube formation was quantified based on the results of (**A**) for MyHC-positivity, differentiation indices, fusion indices, and myotube widths. Results are expressed as the means ± SEMs (*n* = 3; *** *p* < 0.001). ns: no significance.

Since miR-665-3p suppressed the expression of myogenic factors, we next investigated whether miR-665-3p hinders myoblast differentiation and myotube formation. After transfection with scRNA, siTWF1, the miR-665-3p mimic, or antimiR-665, myoblasts were incubated for five days in DM to induce differentiation (Figure 6A,B). Differentiation and myotube formation were then examined by immunocytochemistry using a MyHC antibody, followed by image analysis. Knockdown of TWF1 using siTWF1 significantly impeded myogenic differentiation, as evidenced by reductions in MyHC-positive area percentage, differentiation indices, fusion indices, and myotube width (Figure 6A,B). Notably, transfection with the miR-665-3p mimic significantly inhibited MyHC-positive areas and also resulted in thinner myotubes with less than six nuclei and lower differentiation and fusion indices (Figure 6A,B). To confirm the role of miR-665-3p in myogenic differentiation, myoblasts were cotransfected with antimiR-665 or scRNA, and, as was expected, antimiR-665 dramatically abolished the inhibitory effects of the miR-665-3p mimic on myoblast differentiation and myotube formation (Figure 6A,B). These results provide robust evidence that miR-665-3p impairs the myogenic differentiation and myotube formation of C2C12 myoblasts.

## 4. Discussion

Although accumulating evidence indicates miRNAs play critical roles in skeletal muscle development and regeneration [26,27], it is unclear how miRNAs induced by SFA or obesity influence the myogenic differentiation of progenitor cells. This study reveals the impacts of SFA-inducible miR-665-3p on TWF1 expression, mechanotransduction, and myogenic differentiation. Our findings provide novel insights regarding the mechanism underlying TWF1 expression and myogenesis in the background of obesity. Specifically, they show (i) PA reduces TWF1 expression and induces miR-665-3p expression in C2C12 myoblasts; (ii) MiR-665-3p directly targets the 3′UTR of *TWF1*; and (iii), more importantly, miR-665-3p induction inhibits the myogenic differentiation of progenitor cells by activating YAP1 via F-actin accumulation, subsequently promoting cell cycle progression and proliferation. Accordingly, this study shows that SFA-inducible miR-665-3p contributes to the epigenetic regulation of TWF1 and impairs myogenic differentiation via the F-actin/YAP1 axis.

The differentiation of diverse progenitor cells has been reported to be suppressed by SFA [4,40]. In regards to the mechanism by which SFAs impair myogenic differentiation, the present study shows, for the first time, that PA suppresses TWF1 expression and suggests this suppression may be an etiology of muscle wasting in obesity. TWF1 is a highly conserved protein of the actin-depolymerizing factor homology family [41] and an essential component of the actin cytoskeleton dynamics due to its ability to cap the barbed ends of F-actin and sequester the G-actin monomer [42,43,44,45]. Accordingly, the induction of TWF1 reduces F-actin content by promoting filament depolymerization [46], whereas the knockdown of TWF1 facilitates F-actin formation and increases cytoskeletal aberrations [42,43,44,45]. The present study shows that the depletion of TWF1 stimulated the nuclear translocation of YAP1 and thus promoted the proliferation of myogenic progenitor cells, consequently suppressing myogenic transcription factor expressions, differentiation, and myotube formation in myoblasts, which concurs with a previous report [16]. Since SFA-induced TWF1 suppression is regarded as contributing significantly to impaired myogenic differentiation, our observations imply that decreased TWF1 expression contributes to the etiology of sarcopenic obesity by coupling fat accumulation and muscle wasting.

Then what mechanism is responsible for the inhibition of TWF1 by SFA in myoblasts? Because target prediction analysis indicated that TWF1 is a potential target of SFA-inducible miR-665-3p, we focused on the epigenetic regulation of TWF1 expression by miR-665-3p (Figure 2). Hsa-miR-665-3p is 20 nucleotides long, located on 14q32.2, and belongs to the DLK1-DIO3 miRNA cluster [28]. Moreover, recent studies have reported that miR-665-3p is significantly upregulated in senescence and in the presence of mitochondrial dysfunction and oxidative stress, which are all closely associated with muscle atrophy and wasting [34,35,36]. Furthermore, increases in miR-665-3p levels were observed in the livers of high-fat diet-fed (HFD) mice, primary hepatocytes treated with palmitic acid, and HFD xenografts [28,29,31]. In addition, we previously found that miR-665-3p was induced in the livers of HFD mice [30] and PA-treated HepG2 hepatocytes [31]. In the current study, PA dramatically enhanced miR-665-3p expression in myoblasts, which indicated that PA alone could increase miR-665-3p expression in myoblasts. However, further research is required to understand how SFA increases the expression of miR-665-3p. More importantly, the study shows that miR-665-3p inhibited TWF1 protein expression by directly targeting the 3′UTR of *TWF1*, which is the first report that certain miRNAs can modulate the actin-binding protein TWF1 via epigenetic regulation. The effect of miR-665-3p on TWF1 expression suggests that the molecular mechanism in myogenic differentiation could be regulated by specific miRNA, which targets the actin-binding protein.

During myogenesis, myoblast proliferation is inversely coordinated with differentiation to myotubes; hence, cell cycle arrest is required to initiate myoblast differentiation [2]. In this aspect, it is suggested that the inhibition of myogenic differentiation by miR-665-3p, found in the present study, was ascribed to the increased proliferation of progenitor cells. This raises the question of how miR-665-3p promotes cell cycle progression and proliferation in myoblasts. Diverse processes associated with cell cycle, proliferation, and myogenic transcriptional activation in progenitor cells involve timely consorted cytoskeletal changes, such as cytoskeleton assembly, disassembly, and rearrangement [6,47,48]. The actin cytoskeleton plays a critical role in mechanotransduction by regulating YAP1 in the Hippo signaling pathway [49]. YAP1 contains multiple phosphorylation sites, which are phosphorylated by LATS1/2 kinases [50]. F-actin rearrangement in response to mechanical and cytoskeletal cues can cause changes in the localization and activity of Hippo pathway components, such as scaffolding the protein angiomotin (AMOT) and the adaptor protein Ajuba. In the absence of F-actin, AMOT and Ajuba form a complex with LATS1/2 and promote their activation, leading to the phosphorylation, cytoplasmic sequestration, and proteasomal degradation of YAP1. Conversely, when F-actin levels increase, it interacts directly with AMOT and Ajuba, disrupting their interaction with LATS1/2 and promoting the nuclear translocation of YAP1 [51,52,53]. While we did not show a direct effect of miR-665-3p on LATS1/2 activity in the current study, we showed that miR-665-3p dramatically increases F-actin by suppressing TWF1 expression. Consequently, F-actin reduces YAP1 phosphorylation and facilitates YAP1 nuclear translocation, and, thus, increased cell proliferation and cell cycle progression eventually impede myoblast differentiation. Similar to our findings, miR-665-3p has been reported to play an important role in oncogenesis by regulating proliferation and survival [28,54,55].

## 5. Conclusions

This is the first study to demonstrate the downregulation of TWF1 expression by SFA, implying that this could play a role in the pathogenesis of sarcopenic obesity by connecting muscle wasting and fat deposition. Furthermore, the observed effects of miR-665-3p on TWF1 expression, mechanotransduction, cell proliferation, and myogenic differentiation provide clues as to how SFAs modulate myogenesis. In conclusion, SFA-inducible miR-665-3p suppresses TWF1 epigenetically and inhibits myogenic differentiation by promoting myoblast proliferation via the F-actin/YAP1 axis. Thus, our results suggest the possibility that the underlying mechanisms of actin remodeling and skeletal muscle homeostasis are epigenetically regulated in obesity.

## Data Availability

The data presented in this study are available on request from the corresponding author.

## Supplementary Materials

The following supporting information can be downloaded at: https://www.mdpi.com/article/10.3390/cells12081114/s1, Figure S1. C2C12 cells were cultured in 96-well plates for 24 h, then treated with 100 μM PA or vehicle control in triplicate as described in Materials and Methods.The effect of PA on C2C12 cell viability was determined with a Quanti-Max Cell Viability Assay Kit (Biomax, Seoul, Korea) using a microplate reader at 450 nm after 24 h, 48 h, and 72 h. The values are expressed as the relative ratio, where the intensity of the vehicle control was set to one. Data are presented as means ± SEMs (n = 3). ns: no significance. Figure S2. (A) Schematic images of C2C12 cell differentiation were captured using light microscopy after treatment with PA or vehicle control. (B) The effect of PA on cell population was assessed by nuclei number on DM5. Data are presented as means ± SEMs (n = 3). ns: no significance, Figure S3. To establish a model of diet-induced obesity in mice, male C57BL/6N mice were fed either NFD or HFD for a duration from 6-wk age to 14 wks, as described in the Materials and Method section. The body weight was recorded weekly. After 1 wk of feeding, HFD significantly increased mouse body weights. Data are presented as means ± SEMs (*n* = 9, *, *p* < 0.05; ***, *p* < 0.001; vs. NFD control), Table S1: Oligonucleotide sequences for transfection, Table S2: Primer lists and PCR conditions for *q*RT-PCR, RT-PCR, and cloning, Table S3: Antibodies list, Table S4: Diet composition.
