# Supplementary material for: Induction of miR-665-3p Impairs the Differentiation of Myogenic Progenitor Cells by Regulating the TWF1-YAP1 Axis
Source: Cells. 2023 Apr 8;12(8):1114. doi: 10.3390/cells12081114 (PMC10136822; doi:10.3390/cells12081114)
Supplement: Supplementary file 1 [file cells-12-01114-s001.zip › Figures S1-S3.pdf]

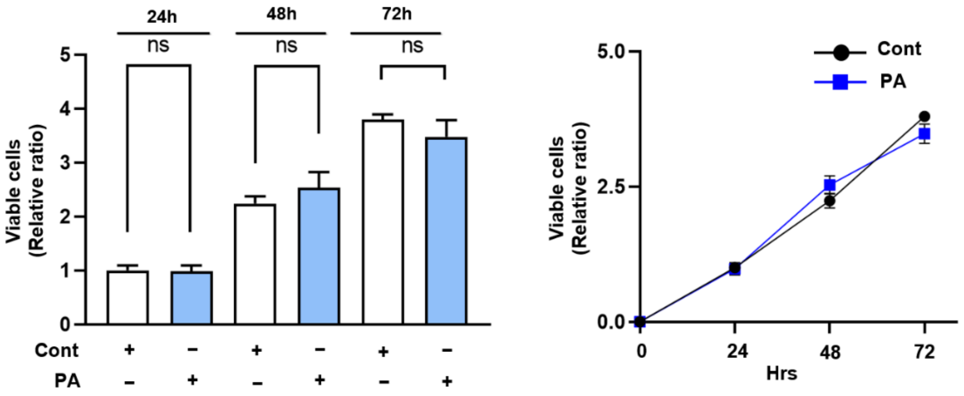

Figure S1. C2C12 cells were cultured in 96-well plates for 24 h, then treated with 100 µM PA or vehicle control in triplicate as described in Materials and Methods. The effect of PA on C2C12 cell viability was determined with a Quanti-Max Cell Viability Assay Kit (Biomax, Seoul, Korea) using a microplate reader at 450 nm after 24 h, 48 h, and 72 h. The values are expressed as the relative ratio, where the intensity of the vehicle control was set to one. Data are presented as means ± SEMs (n =3). ns: no significance.

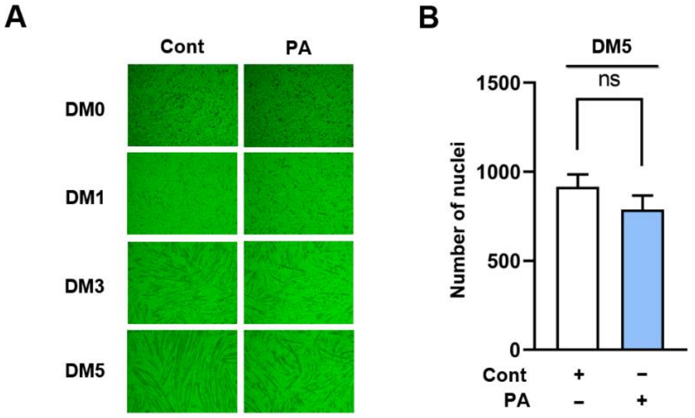

Figure S2. (A) Schematic images of C2C12 cell differentiation were captured using light microscopy after treatment with PA or vehicle control. **(B)** The effect of PA on cell population was assessed by nuclei number on DM5. Data are presented as means ± SEMs (n =3). ns: no significance.

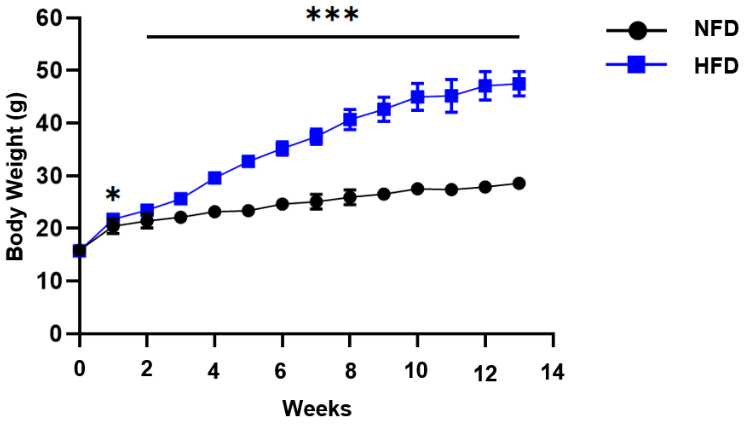

Figure S3. To establish a model of diet-induced obesity in mice, male C57BL/6N mice were fed either NFD or HFD for a duration from 6-wk age to 14 wks, as described in the Materials and Method section. The body weight was recorded weekly. After 1 wk of feeding, HFD significantly increased mouse body weights. Data are presented as means ± SEMs (n =9, *, $P<0.05$; ***, $P<0.001$; vs. NFD control).
