# Supplementary material for: Induction of miR-665-3p Impairs the Differentiation of Myogenic Progenitor Cells by Regulating the TWF1-YAP1 Axis
Source: Cells. 2023 Apr 8;12(8):1114. doi: 10.3390/cells12081114 (PMC10136822; doi:10.3390/cells12081114)
Supplement: Supplementary file 1 [file cells-12-01114-s001.zip › Table S1-S4.pdf]

Table S1. Oligonucleotide sequences for transfection

Gene	Primer sequence (5′-3')
scRNA (control RNA)	UCACAACCUCCUAGAAAGAGUAGA
TWF1 siRNA (siTWF1)	CGUUACCAUUUCUUUCUGUUU
miR-665-3p	ACCAGGAGGCUGAGGCCCCU
antimiR-665	AGGGGCCUCAGCCUCCUGGU

Table S2. Primer lists and conditions for *q*RT-PCR, RT-PCR, and cloning

(A) Mouse primer lists for *q*RT-PCR and RT-PCR

Gene	Primer sequence (5′-3')		Product size	Annealing Temperature	Concentration		Cycle
					cDNA	Primer	
U6	F.P	CTCGCTTCGGCAGCACA	94	58	2 ng/µl	0.5 µM	40
	R.P	AACGCTTCACGAATTTGCGT					
PCNA	F.P	GAACCTGCAGAGCATGGACTC	201	58			
	R.P	GGTGTCTGCATTATCTTCAGCCC					
CCNB1	F.P	GAGCTATCCTCATTGACTGG	125	58			
	R.P	CATCTTCTTGGGCACACAAC					
CCND1	F.P	ACCAATCTCCTCAACGACCG	228	58			
	R.P	ACGGAAGGGAAGAGAAGGG					

(B) Primer lists for wild-type and mutant 3'UTR cloning

Gene	Primer sequence (5′-3')		Product size	Annealing Temperature	Concentration		Cycle
					cDNA	Primer	
TWF1*wt*	F.P	CCTAGAGAGATTTTGAGCCTC	649	58	2 ng/µl	0.5 µM	35
	R.P	ATTCTGGAATGTCCATTTAC					
TWF1*mut*	F.P	CCTAGAGAGATTTTGAGCCTC	502				
	R.P	TGGTCTGCTCGTCGATTCTTAATGC					
	F.P	GCATTAAGAATCGACGAGCAGACCA	178				
	R.P	ATTCTGGAATGTCCATTTAC					

Table S3. Antibodies list

Antibody	Type	Targeted species	Manufacturer	Cat. No.	Dilution ratio*
TWF1	Polyclonal	Rabbit	Proteintech, Rosemont, Illinois, USA	11732-1-AP	1:5,000
MyHC	Monoclonal	Mouse	DSHB, Iowa, IA, USA	MF20	1:1,000
MyoD	Monoclonal	Mouse	Santa Cruz Biotechnology, Dallas, TX, USA	sc-377460	1:1,000
MyoG	Monoclonal	Mouse	Santa Cruz Biotechnology, Dallas, TX, USA	sc-12732	1:1,000
YAP1	Monoclonal	Rabbit	Cell Signaling Technology, Danvers, MA, USA	14074S	1:10,000
p-YAP1	Polyclonal	Rabbit	Cell Signaling Technology, Danvers, MA, USA	4911S	1:10,000
Lamin B2	Monoclonal	Rabbit	Abcam, Cambridge, United Kingdom	ab151735	1:2,500
α-Tubulin	Monoclonal	Mouse	DSHB, Iowa, IA, USA	12G10	1:2,000
β-actin	Monoclonal	Rabbit	Sigma-Aldrich Chemical, St. Louis USA	A2066	1:10,000
Antibodies HRP-linked anti-rabbit IgG			Cell Signaling Technology, Danvers, MA, USA	#7074	1:10,000
Goat anti-mouse(H+L)			Invitrogen, Thermofisher Scientific, Waltham, MA, USA	#32430	1:2,000

*All blots were visualized using a Femto reagent (Thermofisher Scientific).

Table S4. Diet composition

(A) Normal fat diet (Purina Lab), Cat. 38057

Calories (%)		Fat component of total fat (gram%)		
		Saturated fatty acid	Unsaturated Fatty acids	Unidentified fat
Fat	12.41%	Arachidonic Acid (4.42%)	Omega-3 fatty acid (24.55%)<br>Linoleic acid (24.15%)<br>Linolenic acid (2.65%)	Unidentified (44.23%)
Carbohydrate	63.07%			
Protein	24.52%			
Total	100%	4.42% of total fat	51.35% of total fat	44.23% of total fat

(B) High fat diet (Research DYETS), Cat. D12492

Calories (%)		Fat component of total fat (gram%)	
		Saturated fatty acid	Unsaturated Fatty acids
Fat	60%	Palmitic acid (19.64)<br>Stearic acid (10.59%)<br>Myristoleic acid (1.10%)<br>Lau acid (0.8%)<br>Margaric acid (0.35%)<br>Arachidic acid (0.16%)<br>Pentadecanoic acid (0.08%)<br>Capric acid (0.04%)	Oleic acid (33.68%)<br>Linoleic acid (28.37%)<br>Linolenic acid (2.04%)<br>Arachidonic acid (1.78%)<br>Palmitoleic acid (1.33%)<br>Docosapentaenoic acid (0.78%)
Carbohydrate	20%		
Protein	20%		
Total	100%	32% of total fat	68% of total fat
